# Academic data derived from a university e-government analytic platform: An educational data mining approach

**DOI:** 10.1016/j.dib.2023.109357

**Published:** 2023-07-02

**Authors:** Konstantinos Chytas, Anastasios Tsolakidis, Evangelia Triperina, Nikitas N. Karanikolas, Christos Skourlas

**Affiliations:** University of West Attica, Agiou Spiridonos 28, Egaleo 122 43, Greece

**Keywords:** Educational Data Mining, Blended Learning, Learning Analytics, Online Services, Knowledge Discovery, Information System

## Abstract

The article describes the academic data, which derived from a University E-government analytic platform, which supports the facilitation of blended learning in a Greek University during and after the COVID19 outbreak [1,2]. University e-government services refer to a set of information systems that facilitate the functionalities of the University and enable the management of the underlying information [3,4]. These educational, research and managerial services, also called U-EGOV, follow the four stages of e-government (Presence, Interaction, Transaction, Transformation) [5]. In the presented approach, the data was aggregated from the university services with an automated process and includes all the individual U-EGOV services, that is the synchronous and asynchronous educational platforms, the teleconferencing tool, etc. The dataset created contains information about the courses, the assignments, the grades, the examinations, as well as other significant academic elements of the synchronous and the asynchronous education that takes place in the University. The analysis spans from the spring semester of the academic year 2019–2020, the winter semester of the academic year 2020–2021 to the spring semester of 2020–2021 (three semesters in total). The sample consists of 4800 records and after the preprocessing 4765 records (statistics of courses attended by students) which include 1661 unique students within the university in twenty (20) courses. We have followed an educational data mining approach on the collected data by utilizing an automated data aggregation mechanism to gather data for the selected courses, in order to enhance the learning process and the quality of services. The dataset can be reused: i) as a reference point to measure the quality of the academic outputs and its progress through the years and ii) as a basis for similar analysis in other Higher Educational Institutions (HEIs).

**Specifications Table**

**Table:** 

Subject	Education

Specific subject area	Data generated using educational technologies at tertiary education level.
Type of data	Table (csv format)
How the data were acquired	The data was accumulated by the educational web services of the University using an automatic data collection module. The information stems from manifold sources, and more specifically from all the constituents of the U-EGOV services, including the synchronous and asynchronous systems which are employed for the educational activities within a Greek University, which correspond to the e-learning system, the blending learning tools and the teleconferencing applications. Following the inclusion of the basic information required by the system, the automated data aggregation process occurs, and the related data for the imported courses are collected.
Data format	Raw Analyzed Filtered
Description of data collection	The data was collected from the digital infrastructure of the University. The dataset provided for this research is constructed using data from two different platforms: The MS Team platform and the Open e-Class Platform. Our dataset was extracted from MS Teams and involves 13 attributes related to the courses’ meetings that lecturers participate in. Our next source of data is the (open) e-Class Platform which includes statistics about the students’ behavior on the platform. Data becomes completely anonymized in the presented dataset, as no reference to the participant is kept. Only a newly auto-increment ID per student is used. Moreover, to provide anonymization to the professors, we have chosen to keep the course and the corresponding professor hidden in the dataset. As described previously, the aim of our approach is to build a model which could identify students at risk of failing or dropout a course and to prevent that from happening by predicting and informing the stakeholders at an early stage. The final dataset consists of 32 attributes (34 if we count the two hidden fields, that is the professor and the course). It requires preprocessing before parsing the data to the classification algorithms. As the profiles of the students are created based on data retrieved from the various individual information systems of the University, a minor portion of students’ profiles have incomplete data, which are replaced by the average or the median value per case. Furthermore, when a specific subsystem is not used by the academic community, the respective attributes have null values for the students, therefore these attributes are omitted from the dataset. After the substitution of the missing values and the elimination of null values, the data preparation process takes place, and the redundant attributes are identified using Pearson's correlation.
Data source location	• Institution: University of West Attica • City/Town/Region: Aigaleo, Athens • Country: Greece Population sample data provided in this article was obtained at the University of West Attica.
Data accessibility	Primary data was derived from http://e-class.uniwa.gr and https://teams.microsoft.com/. Secondary data is publicly available and were deposited on: Repository name: Academic Data Derived from Blended Learning Data identification number: 10.17632/z62gdty498.1 Direct URL to data: https://data.mendeley.com/datasets/z62gdty498
Related research article	Chytas, K., Tsolakidis, A., Triperina, E. and Skourlas, C. (2022), “Educational data mining in the academic setting: employing the data produced by blended learning to ameliorate the learning process”, Data Technologies and Applications, Vol. ahead-of-print No. ahead-of-print, pp. 1-19. https://doi.org/10.1108/DTA-06-2022-0252

**Value of the Data**

• The data generated by a University E-government analytic platform, that facilitates blended learning, is used as feedback to enhance the educational process. It is of vital importance for all the involved stakeholders.
• The stakeholders that benefit from this dataset include academics and students.
• The data presented in this article can be used to analyze academic performance, to check the areas that need to be enhanced and to implement the required modifications to the educational process. The data can also show how educational performance has developed through the years and can be used as a reference point for other similar studies for tertiary level.

## 1 Objective

Nowadays, there is a profusion of data generated from compulsory distance education caused by the COVID-19. This data can give us new perspectives about the indicators that affect the academic performance of the students. By applying methods from Educational Data Mining and Learning Analytics, we provide a robust and replicable technique that can assist the regulation and enhancement of the educational pathways of students. The main objective for creating this dataset was the need to improve the educational process by taking advantage of the already offered education and its outputs. The dataset not only includes information about synchronous and asynchronous education, as well as log data of the students and professors, but also performance data of the students in the various courses. In addition to the previously published research article, this article offers a more detailed presentation of the dataset itself.

## 2 Data Description

The data stems from the university web services that facilitate synchronous and asynchronous learning, comprising the teleconferencing system (MSTeams) and the Learning Management System (e-Class) respectively, for selected courses of the Department of Informatics. In our approach, we have developed a university e-government analytics platform to support the integration of various University e-Government systems, aiming to collect all the primary data produced during the educational activities of the university. Our prototype analyzes the primary data using machine-learning algorithms, produces secondary data and delivers the results of the analysis to the involved stakeholders. Moreover, we have applied a digital archiving mechanism in order to store all the generated data through our e-government platform. Using digital archiving of the primary data [6], we provide the stakeholders with the opportunity to access all the accumulated information in one place and to retrieve the required data upon request. By maintaining digital archiving, the data of the previous academic years is also available, which is crucial for the data analytic process.

**Fig. 1:**
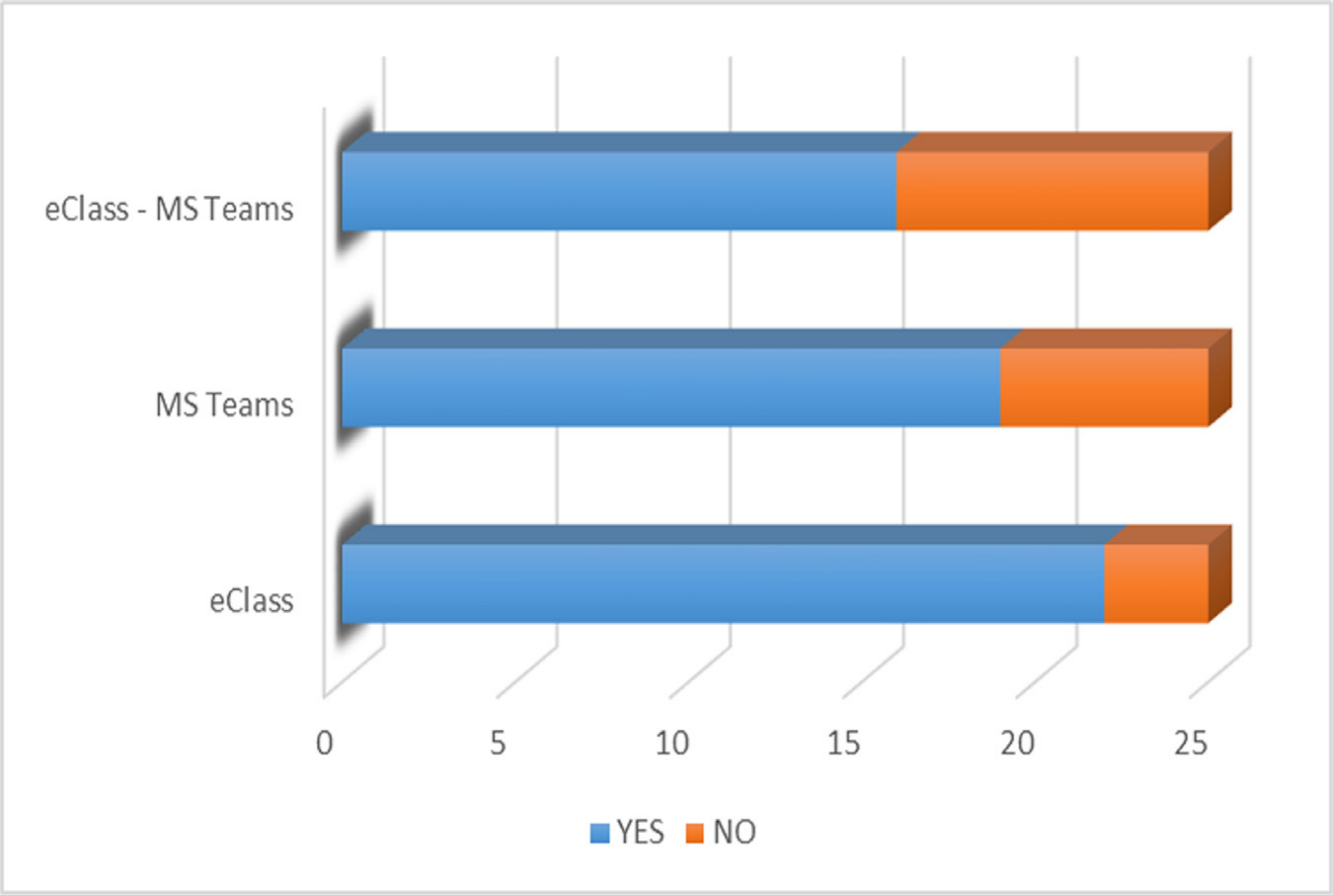
Use of E-learning tools in Greek higher education.

The LMS was employed for the distribution of learning material and the submission of exercises and assignments. It must be mentioned that the github assignments (GitHub Classroom (GHC)) are factored in the assignments of the LMS. MSTeams and e-Class are the main e-learning tools employed by the majority of the Greek Universities (Fig. 1), with 25 higher educational institutes in total and based on the information hosted on GUnet (Greek Universities Network) website [7]:

• 23 Greek Universities use Claroline platform (Open e-Class), regardless of the employed synchronous platform.
• 19 use MSTeams, regardless of the asynchronous platform, whereas.
• 16 utilize Claroline and MSTeams combined.

In the following section, the indicators that were used as a basis for our analysis, as well as the fields of the resulting dataset used in our method are thoroughly presented. Due to the limited amount of the automated statistics that are provided by MSTeams and mainly the absence of the direct matching between a meeting and a course, a series of scripts are executed to scan the individual web pages of the platform, to collect data, to store them in the database and to deduce the way that they are connected. The teleconferencing platform was utilized as a substitution for the in-person lectures during the COVID lockdown. The data that was aggregated spans from spring/summer semester of the academic year 2019–2020, the fall/winter semester of the academic year 2020–2021 to the spring/summer semester of 2020–2021, three semesters in total. In our dataset there is information about 20 courses, in which 1661 students participated in both platforms (1900 unique students in e-Class and 1661 unique students in MSTeams) and 30 different educators, including professors and teaching collaborators that teach those courses (Table 1). We have also checked the participation of the students in the respective courses on the asynchronous learning platform (e-Class) during compulsory distance education. Concerning the e-Class, we have analyzed the data collected from its 34 subsystems. The total number of accesses to these individual subsystems is 270059.

**Table 1:** Total records in e-Class.

e-Class	Value
Total Courses	20
Professors and Teaching Collaborators	30
Student Accounts	1900
Subsystems (Documents, Announcements, Exercises, etc.)	34
Total Number of Access to Subsystems	270059

The indicator final mark derived from the e-Class of that particular course (Table 2). The first indicator, after the anonymization process, is a unique identifier instead of the student email.

**Table 2:** Information derived by the *e-Class of the respective course.*

*a/a*	*Attributes*	*Description*
*1*	Student Email which is anonymized in the dataset	Student id (unique identifier)
*2*	Final Mark	Final mark to course

The e-class subsystems are presented in Table 3. For each e-class constituent, we have collected data related to the student activities (use by the students of the course's subsystems). The attributes on which we focused were the following:

• Access Number, which is the number of visits to the subsystem.
• Duration is the time in minutes remaining in the specific component.

**Table 3:** e-Class course statistics.

a/a	*e-Class Subsystem*	*Description*
1	Announcements	Announcements published from the educational staff
2	Units	Thematic Units
3	Documents	Course Material (.pdf, .doc, …)
4	Exercises	On line exercises using Multiple Choice, …
5	Works	Assignments during the semester
6	Glossary	(Not Used)
7	Learning Paths	(Not Used)
8	Groups	Group of Students
9	Ebook	(Not Used)
10	Chat	Group based Chat
11	Questionnaire	Student Opinion Capturing
12	Agenda	(Not Used)
13	Links	Links to external educational material
14	Forum	Student Forum
15	Video	Links to multimedia educational material
16	Blog	(Not Used)
17	Mind Map	(Not Used)

Based on the results of the analysis of the data, some subsystems were not used by the academics.

Table 4, Table 5, Table 6 illustrate information about MSTeams, the platform which facilitated the synchronous education. Moreover, Table 4 depicts the statistics of the selected courses in the before mentioned platform. There is a difference between the amount of student accounts hosted on the two platforms, due to the fact that all the lectures of the courses were recorded and there are several students, which did not participate in any synchronous lecture. Eventually, we limited our case study to the 1661 students that participated in both platforms.

**Table 4:** Total records in MS Teams.

Microsoft & Teams	Amount
Total Number of Accounts	1691
Student Accounts	1661
Faculty Accounts, Professors and Teaching Collaborators	30
Total Courses	20
Total Number of Meetings	288
Total Number of Connections of Participants in meetings	487000

The attributes (Table 5) Meetings Count, Sessions Count and Meetings Duration are parsed from MS Teams, whereas Channel Messages, Reply Messages, Post Messages, Chat Messages, Urgent messages, Total meetings, Meetings organized, Meetings participated, Audio Time and Video Time are aggregated to the system from the MS Teams Statistics (Table 6). The data file shared in the repository (https://data.mendeley.com/datasets/z62gdty498) is a CSV file, where each record represents a unique student. This is a superset of the individual datasets and consists of the indicators described in the following table (Table 7).

**Table 5:** MS teams.

*a/a*	*Attributes*	*Description*
*1*	Meetings Count	The number of meetings that the student participates
*2*	Sessions Count	The number of times connected in meetings
*3*	Meetings Duration	The time in minutes spent in meetings

**Table 6:** MS teams statistics.

*a/a*	*Attributes*	*Description*
*1*	Channel Messages	The number of unique messages that the student posted in a team chat
*2*	Reply Messages	The number of unique reply messages that the student posted in a team chat
*3*	Post Messages	The number of unique post messages that the student posted in a team channel
*4*	Chat Messages	Τhe number of unique messages that the student posted in a private chat
*5*	Urgent messages	Τhe number of urgent messages that the student posted in a chat
*6*	Total meetings	Τhe total number of scheduled and ad hoc meetings a student sent
*7*	Meetings organized	The total number of scheduled and ad hoc meetings a student organized
*8*	Meetings participated	The number of the scheduled and the ad hoc meetings a student participated in
*9*	Audio Time	The total audio time that the student participated in
*10*	Video Time	The total video time that the student participated in

**Table 7:** Resulting data (shared in the repository).

*a/a*	*Attributes*	*Description*
*1*	student_id	The anonymized unique identifier
*2*	semester	The semester to which the data refers to
*3*	AccessNum	The total number of Access to E-class Platform
*4*	AccessTime	The total time spen d to E-class Platform
*5*	AnnouncementsDays	The total number of days connected to subsystem of “Announcements”
*6*	AnnouncementsAccessNum	The total number of Access to subsystem of “Announcements”
*7*	AnnouncementsAccessTime	The total number of time spend to subsystem of “Announcements”
*8*	UnitsDays	The total number of days connected to subsystem of “Units”
*9*	UnitsAccessNum	The total number of Access to subsystem of “Units”
*10*	UnitsAccessTime	The total number of time spend to subsystem of “Units”
*11*	DocumentsDays	The total number of days connected to subsystem of “Documents”
*12*	DocumentsAccessNum	The total number of Access to subsystem of “Documents”
*13*	DocumentsAccessTime	The total number of time spend to subsystem of “Documents”
*14*	ExercisesDays	The total number of days connected to subsystem of “Exercises”
*15*	ExercisesAccessNum	The total number of Access to subsystem of “Exercises”
*16*	ExercisesAccessTime	The total number of time spend to subsystem of “Exercises”
*17*	WorksDays	The total number of days connected to subsystem of “Works”
*18*	WorksAccessNum	The total number of Access to subsystem of “Works”
*19*	WorksAccessTime	The total number of time spend to subsystem of “Works”
*20*	GroupsAccessNum	The total number of Access to subsystem of “Groups”
*21*	Meetings	The number of meetings that the student participates
*22*	Sessions	The number of times connected in meetings
*23*	Duration	The time in minutes spent in meetings
*24*	ChannelMessages	The number of unique messages that the student posted in a team chat
*25*	ReplyMessages	The number of unique reply messages that the student posted in a team chat.
*26*	PostMessages	Τhe number of unique messages that the student posted in a private chat
*27*	ChatMessages	Τhe number of unique messages that the student posted in a private chat
*28*	MeetingsOrganized	The total number of scheduled and ad hoc meetings a student organized
*29*	MeetingsParticipated	The number of the scheduled and the ad hoc meetings a student participated in
*30*	AudioTime	The total audio time that the student participated in
*31*	VideoTime	The total video time that the student participated in
*32*	GradeFinal	The final grade of the student in the selected course (0-10), in which the grades lower than 5 were substituted by the indication “fail”

**Fig. 2:**
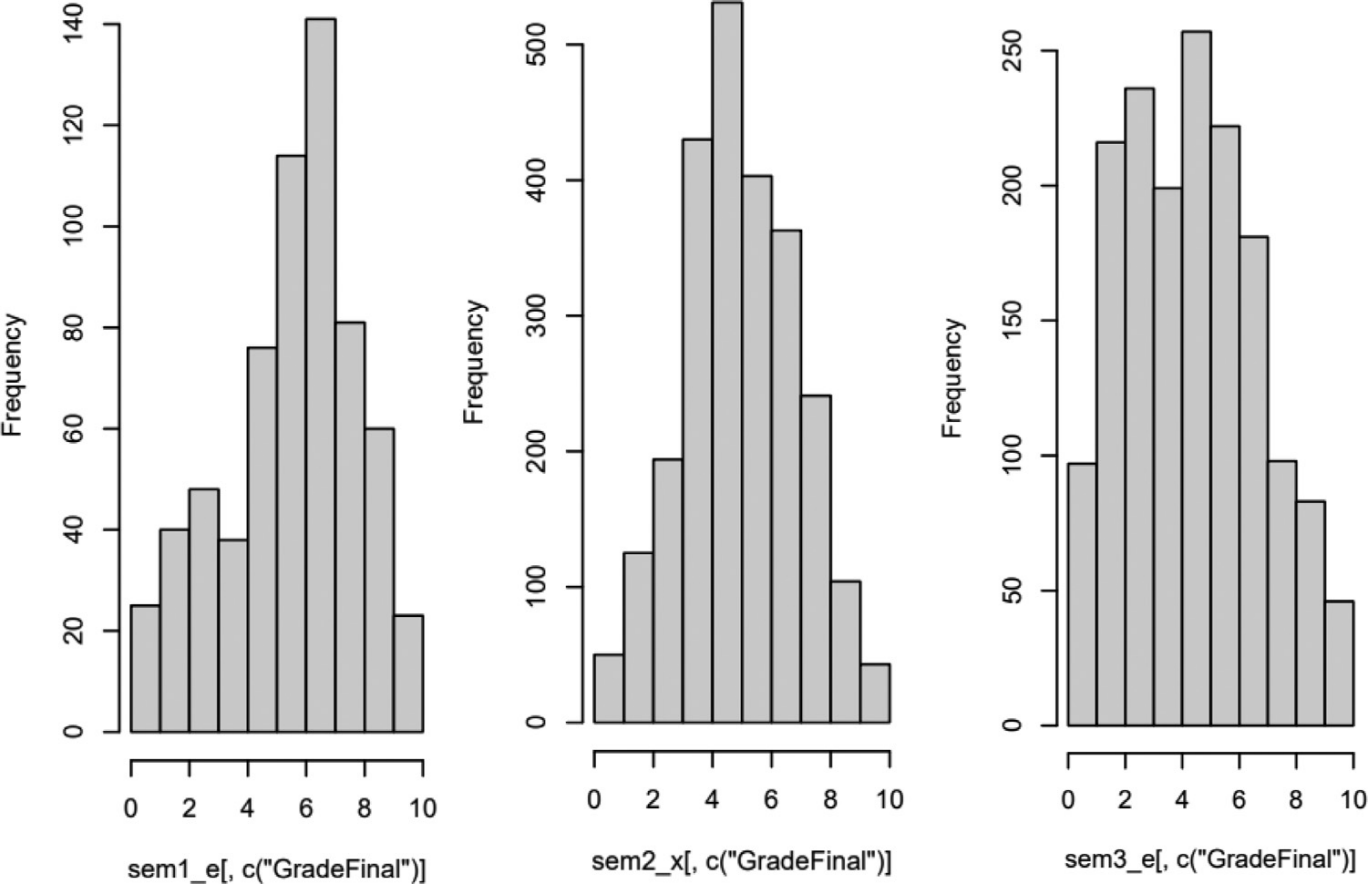
Histograms for the semesters 03/2020-07/2020, 09/2020-02/2021 and 03/2021- 07/2021, respectively.

**Fig. 3:**
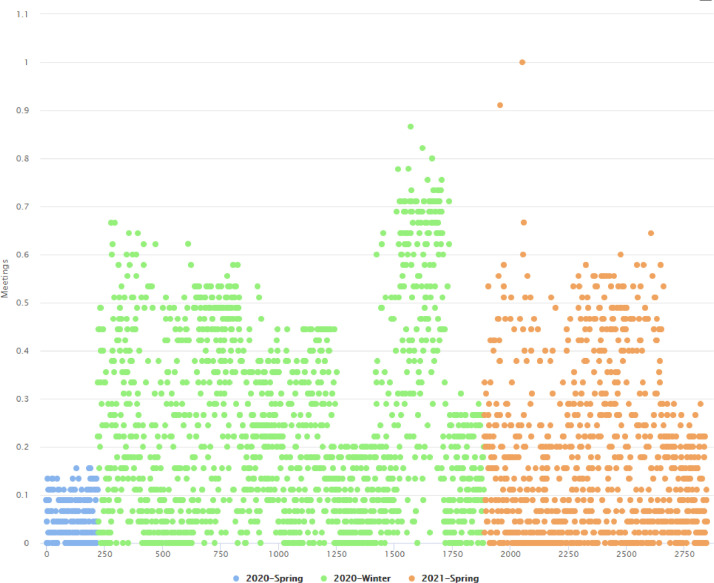
Participation to synchronous meetings during the semesters.

We have studied the impact of COVID on the educational process and the way that the individual factors impact the final outcome of education. Furthermore, we have examined the level in which the employment of the teleconferencing platform assisted in that direction and whether or not its utilization affected the behavior of the students in relation to the teleconferencing platform. Initially, we examined 3 semesters. As histograms show (Fig. 2), in our dataset, there is a normal distribution concerning the attribute of final grade, in the three before mentioned semesters. In order to evaluate the impact of synchronous learning during the educational process we are going to measure the correlation between each variable. Because we are interested in the students who participate in the meetings, we are going to withdraw the records of the students that did not participate in any meeting. Due to the different range of the values, we normalize the data to fit to the range 0-1. As we can see in the following figure (Fig. 3), during the first semester, the participation to the synchronous meetings is limited, but through the next semesters there is a rapid increase of the participations. A possible explanation could be that academia was not fully ready to transition to the synchronous platform, then in the subsequent semesters both students and professors were acclimatized.

**Fig. 4:**
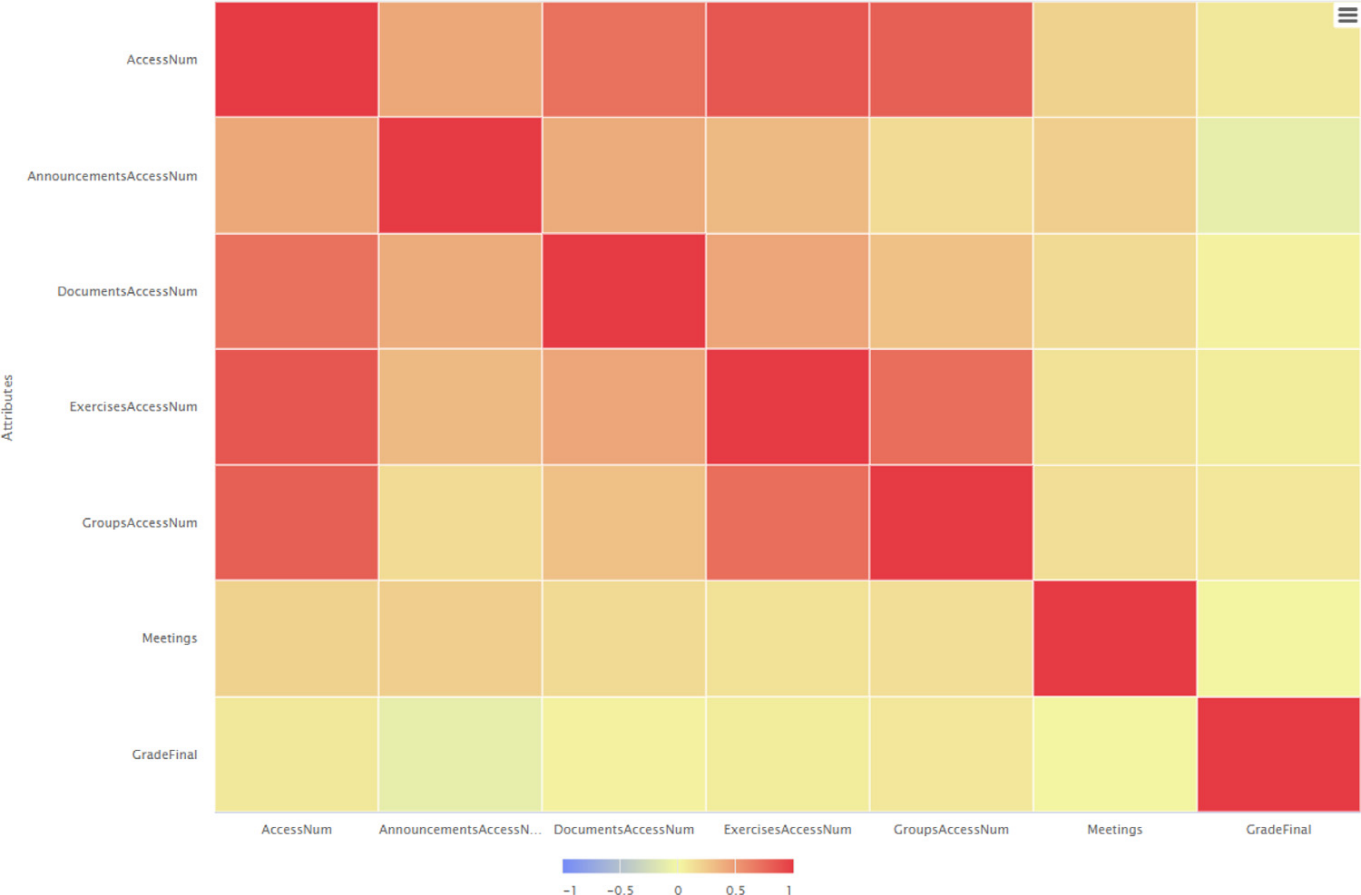
Correlation matrix spring/summer semester 2020: correlation of attributes.

**Fig. 5:**
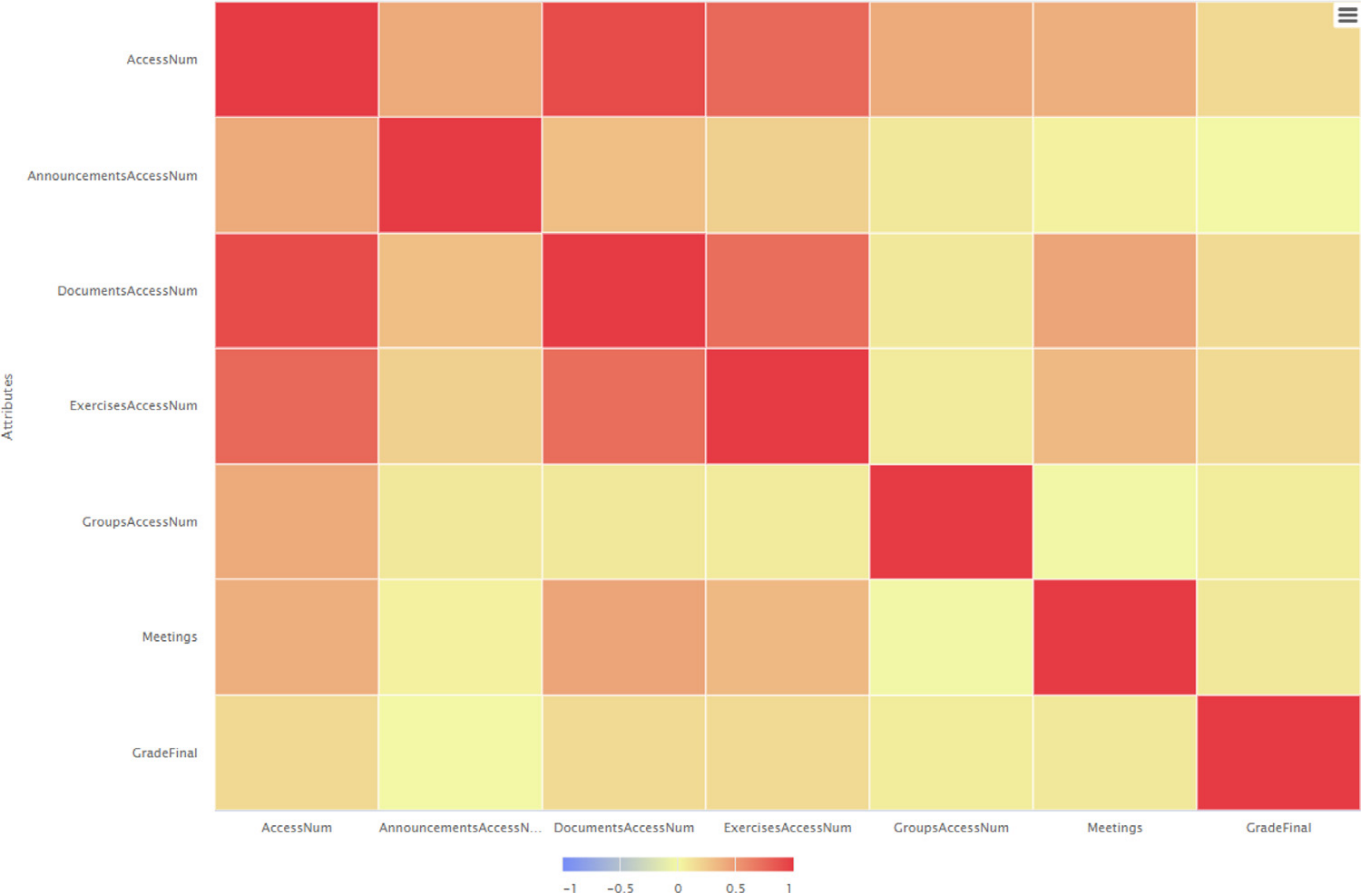
Correlation matrix winter semester 2020: correlation of attributes.

**Fig. 6:**
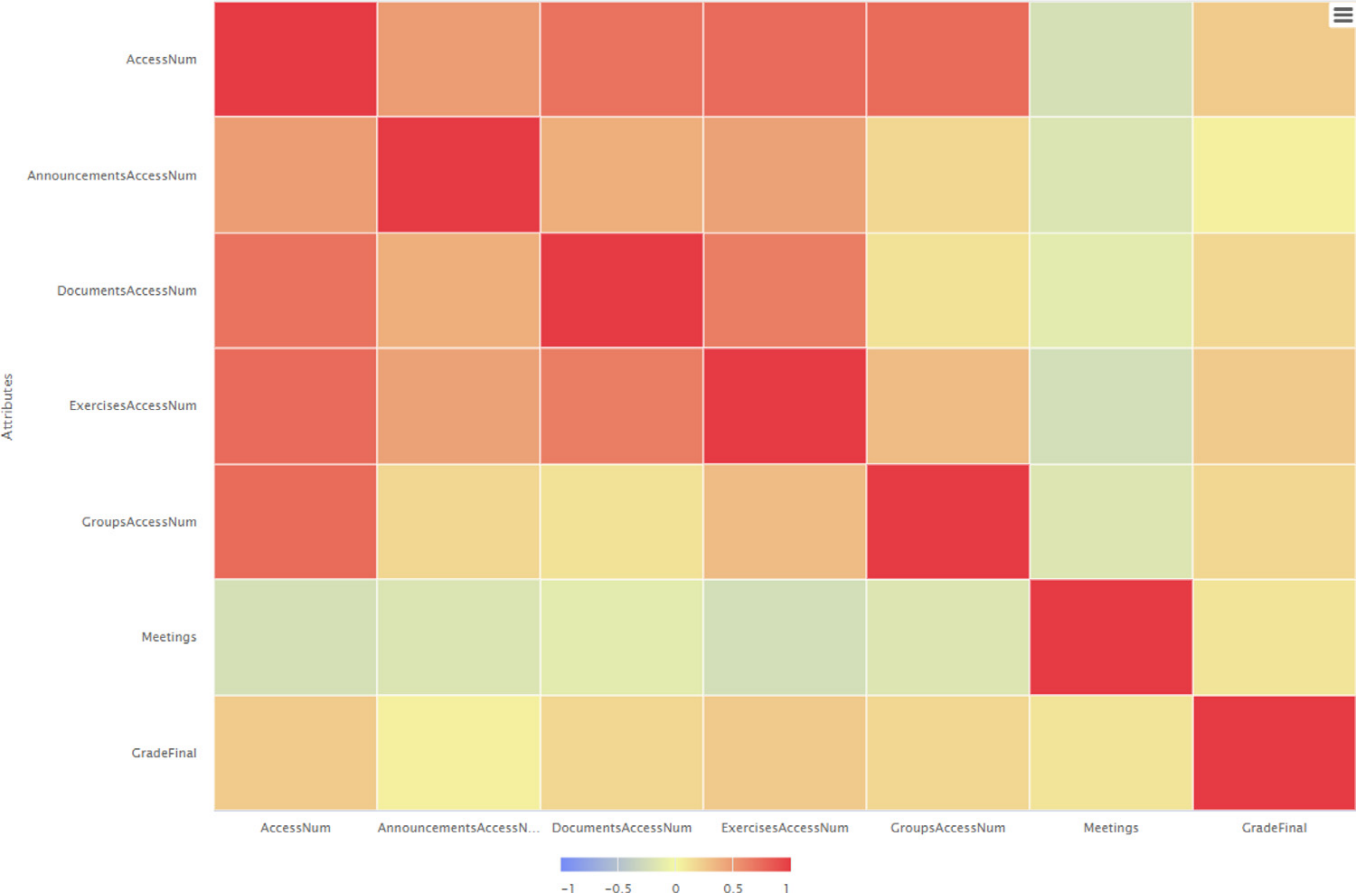
Correlation matrix spring/summer semester 2021: correlation of attributes.

According to the correlation matrices presented in Figs. 4, 5 and 6, a low correlation among the participation in meetings and grades was evident initially, whereas during the next semester the correlation degree has been increased and finally in the third semester the correlation gets negative values. Also, all the correlation matrixes support that e-Class values are highly correlated with final grade of students.

**Fig. 7:**
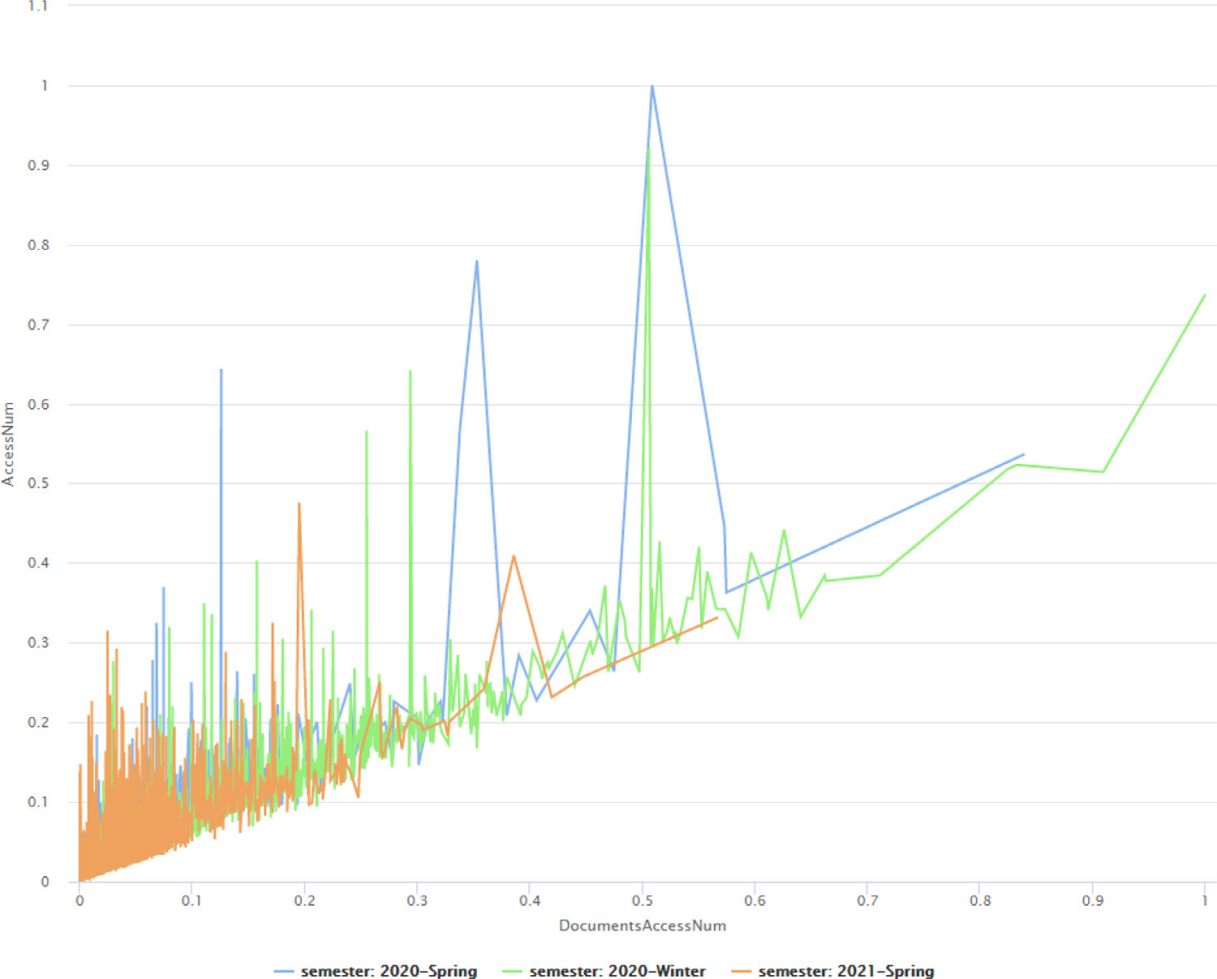
Behavior of the students towards the asynchronous platform.

Finally, in Fig. 7, we examine the behavior of the students towards the asynchronous platform taking into consideration its two most representative attributes, namely AccessNum and DocumentAccessNum. The two attributes are highly correlated, and the students seem to have the same values in both variables. However, in the third semester the visits of the students to the asynchronous platform were reduced.

To sum up, we see that the use of e-Class has a crucial role in the final grade during the academic periods. Initially the synchronous platform (MSTeams) is used with limited participation of the students, then in the next semester the students got used to the e-learning technologies and their grade is also influenced from the level of usage, whereas at third semester the use of e-Class seems to diminish.

In our approach, we have developed a University E-government analytic platform, which collects data from the existing University information systems to better support the educational process. The aim of our prototype is to provide access to all the information produced during the education activities to all the related stakeholders to facilitate insightful and informed decisions.

## 3 Experimental Design, Materials and Methods

This section covers the experimental design that was used in order to create the presented dataset. After the aggregation of the data from the different sources, the experiment on the way that the end result (success or failure) of a course is impacted by the participation and the performance of the students through the semester took place.

As far as it concerns the data collection process, it comprises the accumulation of the data from the involved subsystems, that is the synchronous and the asynchronous e-learning platforms. Following the data aggregation process, the preprocessing of the dataset occurs. Preprocessing of the data is a required step that precedes the parsing of the data to the classification algorithms.

The dataset was extracted and converted into CSV format. Each record depicts a unique student's performance in a single course for a specific semester (student-course-semester). The final dataset after the prepocessing consists of 4765 total records, as described above, which corresponds to 1661 unique students and their performance in twenty (20) courses for three (3) semesters. It must be mentioned that the number of total records depends on what courses the 1661 unique students have selected in each academic period. The students have different combinations of student – course – semester, which serves as a composite key.

The above-described methodological approach combines the scientific fields of educational data mining, machine learning algorithms and visualizations to ameliorate the provided services within the University, by making the most out of the feedback generated from the educational data, which include participation and socialization data and behavioral data.

## Ethics Statements

This study does not involve experiments on humans or animals. This work does not entail gathering information from social media platforms. The only data source used in this work is the involved systems’ relational database, which does not include any user-related social media information. The dataset is not linked to any third-party apps or platforms. The dataset contains no sensitive information. University of West Attica owns this dataset. Data can be used for multiple purposes within the same research domain. Because the data used in this study has already been anonymized, further anonymization before sharing is not required.

## CRediT authorship contribution statement

**Konstantinos Chytas:** Data curation, Software, Methodology, Software, Writing – review & editing. **Anastasios Tsolakidis:** Conceptualization, Methodology, Validation, Writing – review & editing. **Evangelia Triperina:** Writing – original draft, Writing – review & editing. **Nikitas N. Karanikolas:** Conceptualization, Writing – review & editing. **Christos Skourlas:** Supervision, Conceptualization, Writing – review & editing.

## Declaration of Competing Interest

The authors declare that they have no known competing financial interests or personal relationships that could have appeared to influence the work reported in this paper.

## Data Availability

Academic Data Derived from Blended Learning (Original data) (Mendeley Data).
