# Corrigendum to ``Academic data derived from a university e-government analytic platform: An educational data mining approach'' [Data in Brief, Volume 49 (2023) /109357]

**DOI:** 10.1016/j.dib.2023.109494

**Published:** 2023-08-13

**Authors:** Konstantinos Chytas, Anastasios Tsolakidis, Evangelia Triperina, Nikitas N. Karanikolas, Christos Skourlas

**Affiliations:** Department of Informatics and Computer Engineering, University of West Attica, Agiou Spiridonos 28, Egaleo 122 43, Greece

The authors regret < The acknowledgments ``This study has received financial support from the Special Account for Research Grants (ELKE) of University of West Attica.'' have not been included in the Acknowledgments section of the paper. Please add the before mentioned text in the Acknowledgments section.>.

The authors would like to apologise for any inconvenience caused.

